# Systematic review of the measurement properties of indices of prenatal care utilization

**DOI:** 10.1186/s12884-020-2822-5

**Published:** 2020-03-18

**Authors:** Stewart Rowe, Zahra Karkhaneh, Isaiah MacDonald, Thane Chambers, Sana Amjad, Alvaro Osornio-Vargas, Radha Chari, Manoj Kumar, Maria B. Ospina

**Affiliations:** 1 Department of Obstetrics & Gynecology and Medicine, Faculty of Medicine & Dentistry, University of Alberta, Edmonton, Alberta Canada; 2 John W. Scott Health Sciences Library, University of Alberta, Edmonton, Alberta Canada; 3 School of Public Health, University of Alberta, Edmonton, Alberta Canada; 4 Department of Pediatrics, Faculty of Medicine & Dentistry, University of Alberta, Edmonton, Alberta Canada; 5 220B Heritage Medical Research Centre, Edmonton, AB T6G 2S2 Canada

**Keywords:** Prenatal care, Pregnancy, Systematic review

## Abstract

**Background:**

An accurate assessment of the adequacy of prenatal care utilization is critical to inform the relationship between prenatal care and pregnancy outcomes. This systematic review critically appraises the evidence on measurement properties of prenatal care utilization indices and provides recommendations about which index is the most useful for this purpose.

**Methods:**

MEDLINE, EMBASE, CINAHL, and Web of Science were systematically searched from database inception to October 2018 using keywords related to indices of prenatal care utilization. No language restrictions were imposed. Studies were included if they evaluated the reliability, validity, or responsiveness of at least one index of adequacy of prenatal care utilization. We used the COnsensus-based Standards for the selection of health Measurement INstruments (COSMIN) checklist. We conducted an evidence synthesis using predefined criteria to appraise the measurement properties of the indices.

**Results:**

From 2664 studies initially screened, 13 unique studies evaluated the measurement properties of at least one index of prenatal care utilization. Most of the indices of adequacy of prenatal care currently used in research and clinical practice have been evaluated for at least some form of reliability and/or validity. Evidence about the responsiveness to change of these indices is absent from these evaluations. The Adequacy Perinatal Care Utilization Index (APNCUI) and the Kessner Index are supported by moderate evidence regarding their reliability, predictive and concurrent validity.

**Conclusion:**

The scientific literature has not comprehensively reported the measurement properties of commonly used indices of prenatal care utilization, and there is insufficient research to inform the choice of the best index. Lack of strong evidence about which index is the best to measure prenatal care utilization has important implications for tracking health care utilization and for formulating prenatal care recommendations.

## Introduction

Routine prenatal care is a series of regular contacts between a health care provider and a pregnant woman at scheduled intervals that occur between the confirmation of pregnancy and the initiation of labour. The primary goal of these encounters is to deliver effective screening, preventive (education), and treatment interventions that seek to improve health outcomes for both the mother and the newborn. Prenatal care also aims to address behavioural risk factors, support women’s medical, social and psychological needs, and coordinate actions for labour and delivery [1].

The American College of Obstetrics and Gynecology (ACOG) recommends visiting every 4 weeks for the first 28 weeks of pregnancy followed by bi-weekly visits up to 36 weeks. After 36 weeks, weekly visits are advised [2]. Recommendations about timing of initiation and number of routine prenatal care visits for uncomplicated pregnancies differ across jurisdictions, reflecting local contexts, economic, and health policy factors [3, 4] and shifting patterns in the schedules for the frequency and interval between prenatal visits [5–7]. Evidence from systematic reviews indicate inconsistencies and lack of consensus on the optimal components of routine prenatal care (i.e., content, frequency and timing of visits) that underlie its effectiveness to avoid adverse outcomes for mothers and their newborns [8].

A variety of methods have been used in past research to determine adequacy of prenatal care in low-risk pregnancies. Over the last two decades, several scoring systems for prenatal care utilization have been developed, each employing different algorithms: the Kessner Index [9], the Kotelchuk Index –also known as Adequacy Perinatal Care Utilization Index (APNCUI)– [10], the Graduated Prenatal Care Utilization Index (GINDEX) [11], the Revised-Graduated Prenatal Care Utilization Index (R-GINDEX) [12], and the US Public Health Service Expert Panel on Prenatal Care (PHS/EPPC) [13]. In general, these indices are founded on two variables: the initiation of prenatal care (i.e., timing of visits related to the weeks of gestation or trimester in which prenatal care is initiated), and the number of prenatal care visits received throughout pregnancy (i.e., frequency). Ultimately, the indices classify prenatal care utilization into distinct categories based on these two variables. Classification of adequacy of prenatal care utilization is likely to be dependent on the index of choice, and misclassifications can potentially lead to systematic differences in the magnitude and direction of the association between prenatal care utilization and maternal and birth outcomes [12, 14, 15].

The comparability of the different prenatal care utilization indices for low-risk pregnancies has not been completely explored. To date, no systematic review has incorporated a comprehensive analysis of the methods by which prenatal care utilization indices have been developed, nor appraised their relative value. A systematic evaluation of the measurement properties of these indices, their relative strengths and weaknesses, and the quality of the evidence that support their use is an essential step to inform the selection of these indices for research and clinical practice. To fill these knowledge gaps, we completed a systematic review of the scientific literature to assess and compare the measurement properties (i.e., validity, reliability, responsiveness) of prenatal care utilization indices.

## Methods

The systematic review was conducted and reported according to the Preferred Reporting Items for Systematic Reviews and Meta-Analyses (PRISMA) statement [16]. The review protocol was registered with the prospective register of systematic reviews (PROSPERO; registration number CRD42017067110). Comprehensive electronic searches of MEDLINE, EMBASE, CINAHL, and Web of Science were conducted from database inception to October 2018 for studies evaluating the measurement properties of prenatal care utilization indices. An information specialist designed and executed the search strategy using selected subject headings and keywords related to prenatal care utilization indices and measurement properties. The MEDLINE search strategy is available in Additional File 1. In addition, subsequent searches in Google Scholar (for web-based materials) and ProQuest Dissertation and Theses (for unpublished work) were conducted and reference lists of potentially relevant articles were examined. There were no language restrictions imposed on this review.

Indices evaluating prenatal care utilization were defined as quantitative tools that evaluated both the initiation of prenatal care and the frequency at which a pregnant woman attends prenatal care services [12] in low-risk pregnancies. Included in the review were primary studies that evaluated the measurement properties of indices of prenatal care utilization*.* There were no restrictions on the study design; however, book chapters, editorials, letters, and in vitro or animal studies were excluded.

**Fig. 1:**
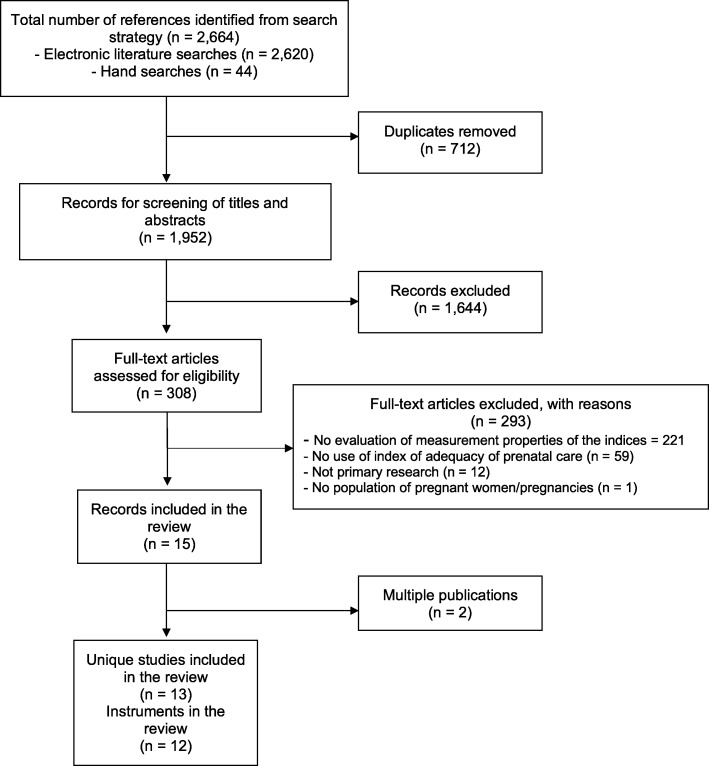
PRISMA study flow for the review

The search strategy generated a list of articles that two reviewers [IM and SR, or SA and MO] screened independently for relevance. Titles and abstracts that were identified as relevant or those that provided insufficient information were pursued for further assessment. The full text of considered articles was again independently reviewed for inclusion [SR, AO, RC, MK and MO], with disagreements resolved through consensus. The final reason for the exclusion of an article was documented in the PRISMA flow chart (Fig. 1).

Two reviewers [SR and MO or IM and MO] independently evaluated the methodological quality of studies assessing the measurement properties of indices of adequacy of prenatal care utilization using the COnsensus-based Standards for the Selection of health Measurement INstruments (COSMIN) checklist [17]. Disagreements were again resolved through consensus. The COSMIN checklist was originally developed to inform evidence-based decisions in the selection of patient-reported outcome measures; however, its use has been extended to other measurement domains such as performance-based outcomes. We have selected for this review items from COSMIN that are likely relevant for the evaluation of prenatal care utilization, a construct that fall within the domain of performance-based measure. Therefore, this review evaluates the methodological quality of studies on measurement properties in the following domains: reliability (i.e., internal consistency, reliability), validity (i.e., content and criterion [predictive and concurrent] validity), and responsiveness (Table 1).

**Table 1:** Measurement properties definitions used in the review

Internal Consistency	Interrelatedness among items.
Reliability	The consistency with which different examiners (inter-rater) or two administrations (test-retest) of a test produce similar ratings in an instrument.
Content Validity	Degree to which an instrument includes all the necessary items to represent the concept to be measured.
Criterion-related: Predictive Validity	The extent to which results of a particular instrument compare with an outcome assessed at a later time.
Criterion-related: Concurrent Validity	The degree to which measurement results are an adequate reflection of another assessment/criterion.
Responsiveness	The ability of an instrument to detect change over time in the construct to be measured

Adapted from Mokkink et al. [18] and Terwee et al. [17]

The COSMIN checklist is frequently used in systematic reviews of indices and measurement instruments, and it is currently the only validated and standardized tool available for this purpose [17]. For the COSMIN assessment, we extracted information on the measurement properties reported on each study and evaluated the quality of the study methodology. COSMIN contains rules for grading the overall methodological quality of studies reporting measurement properties of instruments. For each measurement property, the COSMIN checklist has 5–18 items covering methodological standards that are rated on a 4-point scale (poor, fair, good, excellent). An overall quality score is obtained by taking the lowest ranking for each item [17].

For each index of prenatal care utilization, the overall levels of evidence on each measurement property were synthesized using the data on measurement properties reported in the included studies. If several studies informed the measurement properties of one index, findings were combined based on their number and methodological quality, and the consistency of the results. The level of evidence for the measurement properties of each index was classified according to the following criteria [18]: strong (i.e., consistent findings in multiple studies of good methodological quality *or* in one study of excellent methodological quality); moderate (i.e., consistent findings in multiple studies of fair methodological quality *or* in one study of good methodological quality); limited (i.e., one study of fair methodological quality); conflicting (i.e., conflicting findings); and unknown (only studies of poor methodological quality or no studies at all).

Information on authors, publication year, study design, population characteristics, data sources, and measurement properties evaluated in individual studies were first extracted by one reviewer [SR, SA or IM] and verified for accuracy and completeness by a second reviewer [MO]. Discrepancies between data extraction and verification were sorted through consensus.

## Results

The search strategy identified 2664 citations of which 712 duplicates were removed. Titles and abstracts of the remaining 1952 citations were screened for relevance, yielding to 308 articles judged as potentially relevant for the review. After applying the eligibility criteria to the full text of these and examining redundant publications, 13 unique studies were included in the review (Fig. 1). The complete list of excluded studies is available upon request.

The studies were conducted in the United States [10, 12, 14, 19–23], Brazil [24, 25], Belgium [26], Canada [15], and Spain [27]; and published between 1994 and 2014 (median year of publication 2004; interquartile range [IQR] 1996–2013). Sample sizes varied across studies, ranging from 238 to 591,403 participants (median 8957; IQR 725–147,059). Nine studies used a retrospective cohort design [10, 12, 14, 15, 20–23, 25], two were cross-sectional studies [19, 24], one was a prospective cohort study [26], and one used a case-control design [27]. Characteristics of the study populations, data sources and indices of prenatal care utilization evaluated in the individual studies are described in Table 2.

**Table 2:** Characteristics of the studies investigating measurement properties of indices of prenatal care utilization

Author, Year	Country	Index	Design	Population and Sample Size	Data Source	Overall Quality
Alexander, 1996 [12]	USA	- Kessner - APNCUI - GINDEX - R-GINDEX -PHS/EPPC	Retrospective cohort study	Pregnant women having a singleton live birth in South Carolina *N* = 169,082	Administrative health database (1989–1991)	Fair
Beeckman, 2013 [26]	Belgium	- APNCUI - CTP	Prospective cohort study	Pregnant women seen at medical centres in Brusssels Metropolitan Region *N* = 333	Interview (2008)	Good
da Silva, 2013 [24]	Brazil	- Kessner - APNCUI - IPR/Prenatal Index	Cross-sectional study	Pregnant women seen at primary care services in the municipality of Joao Pessoa *N* = 238	Survey (2010–2011)	Fair
Delgado-Rodriguez, 1996 [27]	Spain	- Kessner - APNCUI	Case-control study	Pregnant women seen at a University hospital in Granada *N* = 632	Chart review and interview (1990–1993)	Fair
Dos Santos, 2013 [25]	Brazil	- Kessner - APNCUI - GINDEX - PHS/EPPC - IPR/Prenatal Index - Carvalho & Novaes Index - Ciari Index - Coutinho Index	Retrospective cohort study	Pregnant women admitted for delivery at public and outsourced maternity hospitals in Greater Metropolitan Vitória *N* = 1006	Chart review and interview (2010)	Fair
Heaman, 2008 [15]	Canada	- APNCUI - R-GINDEX	Retrospective cohort study	Pregnant women having a hospital-based singleton live birth in Winnipeg *N* = 80,989	Administrative health database (1991–2000)	Good
Koroukian, 2002 [19]	USA	- APNCUI	Cross-sectional study	Pregnant women having a singleton live birth in Ohio *N* = 591,403	Administrative health database (1993–1996)	Fair
Kotelchuck, 1994 [10]	USA	- Kessner - APNCUI	Retrospective cohort study	Women with prenatal care information on the birth certificate from the 1980 National Natality Survey *N* = 9941	Survey (1980)	Poor
Kurtzman, 2014 [20]	USA	- APNCUI - LV-APNC Index	Retrospective cohort study	Pregnant women having a singleton live hospital birth in New York State *N* = 58,462	Perinatal Database (2007–2011)	Fair
Penrod, 2000 [21]	USA	- Kessner - APNCUI	Retrospective cohort study	Women with prenatal care information on the birth certificate from the 1980 National Natality Survey *N* = 7973	Survey (1980)	Poor
Perloff, 1997 [22]	USA	- Kessner - APNCUI	Retrospective cohort study	Women with birth certificate data from New York city *N* = 255,884	Administrative health database (1991–1992)	Fair
Rosenberg, 2004 [23]	USA	- APNCUI - Cluster solution	Retrospective cohort study	Women with live birth data from the 1988 National Maternal and Infant Health Survey *N* = 3544	Survey (1988)	Good
VanderWeele, 2009 [14]	USA	- Kessner - APNCUI - GINDEX	Retrospective cohort study	Women with live birth data from the 2003 National Center for Health Statistics Linked Birth and Infant Death Cohort files N = NR	Administrative health database (2003)	Good

*APNCUI* Adequacy of Prenatal Care Utilization Index, *CTP* Content and Timing of Care in Pregnancy, *GINDEX* Graduated Prenatal Care Utilization Index, *IPR* Infrastructure, process, and results, *LV-APNC* Last Visit Adequacy of Prenatal Care Index, *PHS/EPPC* United States Public Health Service Expert Panel on Prenatal Care, *R-GINDEX* Revised-Graduated Prenatal Care Utilization Index, *NR* Not reported, *USA* United States of America

A total of 12 indices of prenatal care utilization were evaluated in the studies. The majority of included studies evaluated more than one index (Table 3). The most frequently evaluated index was the APNCUI (evaluated in all the studies included in the review) followed by the Kessner Index (eight studies [10, 12, 14, 21, 22, 24, 25, 27]). Other indices of prenatal care utilization evaluated for their measurement properties were the GINDEX (three studies [12, 14, 25]), its revised version (i.e., R-GINDEX; evaluated in two studies [12, 15]) and the PHS/EPPC (two studies [12, 25]). Five studies [20, 23–26] evaluated other indices of prenatal care utilization (i.e., Índice IPR/Pré-Natal, Carvalho & Novaes Index, Ciari Index, Coutinho Index, Content and Timing of Care in Pregnancy, a Cluster solution, and the Last Visit Adequacy of Prenatal Care Index [LV-APNC]). Overall, the methodological quality of studies evaluating the measurement properties of these indices was fair, with more recently published studies having a better quality of reporting. Table 3 summarizes the general characteristics of the included indices of prenatal care utilization and the measurement properties that were evaluated in the individual studies.

**Table 3:** Characteristics of Indices of Prenatal Care Utilization Evaluated for their Measurement Properties

Index and Studies	Adequate Start of Prenatal care	Adequate Number of Prenatal Visits	Categories of Prenatal Care	Basis for Standard	Properties Evaluated
APNCUI [10, 12, 14, 15, 19–27]	1–4 mo	11	Intensive Adequate Intermediate Inadequate No care/missing	ACOG	- Reliability - Predictive validity - Concurrent validity
Kessner Index [10, 12, 14, 21, 22, 24, 25, 27]	1–3 mo	9	Adequate Intermediate Inadequate No care/missing	ACOG	- Reliability - Predictive validity - Concurrent validity
GINDEX [12, 14, 25]	1–3 mo	9	Intensive Adequate Intermediate Inadequate No care/missing	ACOG^a^	- Reliability - Predictive validity - Concurrent validity
R-GINDEX [12, 15]	1–3 mo	13	Intensive Adequate Intermediate Inadequate No care Missing	ACOG	- Predictive validity
PHS/EPPC [12, 25]	1–2 mo	7 (multipara); 9 (primipara)	Adequate Intermediate Inadequate No care/missing data	PHS	- Reliability - Concurrent validity
IPR/Prenatal Index [24, 25]	1–3 mo	≥ 6	Adequate Intermediate Inadequate		- Reliability - Predictive validity - Concurrent validity
Carvalho & Novaes Index [25]	1–3 mo	≥ 7	Adequate Inadequate	NR	- Reliability - Concurrent validity
Ciari Index [25]	1–3 mo	11	Good Fair Missing	NR	- Reliability - Concurrent validity
Cluster solution [23]	NR	≥ 12	Six clusters of patterns of prenatal care	NR	- Concurrent validity
Coutinho Index [25]	1–3.5 mo	≥ 6	Adequate Intermediate Inadequate	NR	- Reliability - Concurrent validity
Content and Timing of Care in Pregnancy [26]	1–4 mo	Minimum 80% ratio between visits conducted and expected visits	Appropriate Sufficient Intermediate Inadequate	NR	- Predictive validity
LV-APNC [20]	NR	9	Adequate Plus Adequate Intermediate Inadequate	NR	- Predictive validity

*ACOG* American College of Obstetrics & Gynecology, *IPR* Infrastructure, process, and results, *LV-APNC* Last Visit Adequacy of Prenatal Care Index, *mo* months

^a^Does not follow full ACOG prenatal care visit recommendation for term and post-term births

### Measurement properties and evidence level of the indices of prenatal care utilization

Reliability of the indices was seldom assessed (two studies [21, 25]) while validity was the most frequently evaluated measurement property. Predictive validity was evaluated in seven studies [14, 15, 19, 20, 24, 26, 27], while concurrent validity through head-to-head comparisons among indices was evaluated in six studies [10, 12, 22–25]. The studies did not provide any evidence about the internal consistency, content validity, or responsiveness of any of the 12 indices of adequacy of prenatal care utilization evaluated in this review. An overview of the overall evidence rating for all measurement properties of all indices of prenatal care utilization is provided in Table 4.

**Table 4:** Levels of Evidence for the Measurement Properties of Indices of Prenatal Care Utilization

Index	Internal Consistency	Reliability	Content Validity	Predictive Validity	Concurrent Validity	Responsiveness
APNCUI	?	++	?	++	++	?
Kessner Index	?	++	?	++	++	?
GINDEX	?	+	?	+	++	?
R-GINDEX	?	?	?	+	?	?
PHS/EPPC	?	+	?	?	++	?
IPR/Prenatal Index	?	+	?	+	++	?
Carvalho & Novaes Index	?	+	?	?	+	?
Ciari Index	?	+	?	?	+	?
Cluster solution	?	?	?	?	+	?
Coutinho Index	?	+	?	?	+	?
Content and Timing of Care in Pregnancy	?	?	?	+	?	?
Last Visit Adequacy of Prenatal Care Index	?	?	?	+	?	?

Strong = +++; Moderate = ++; Limited = +; Conflicting +/−; Unknown =?

Reliability was evaluated for eight indices: the APNCUI [21, 25], the Kessner Index [21, 25], GINDEX [25], PHS/EPPC [25], IPR/Prenatal Index [25], Carvalho & Novaes Index [25], Ciari Index [25], and Coutinho Index [25]. Moderate evidence was found for good reliability of the APNCUI and the Kessner indices, while limited evidence supported the reliability of the GINDEX, PHS/EPPC, IPR/Prenatal Index, Carvalho & Novaes Index, and Ciari Index.

Predictive validity was evaluated for the APNCUI [14, 15, 19, 20, 24, 26, 27], the Kessner Index [14, 24, 27], GINDEX [14], R-GINDEX [15], IPR/Prenatal Index [24], Content and Timing of Care in Pregnancy [26], and the LV-APNC [20]. Moderate evidence of predictive validity was found for the APNCUI and the Kessner Index, whereas the evidence was limited for the predictive validity of the GINDEX, R-GINDEX, IPR/Prenatal Index, Content and Timing of Care in Pregnancy and the LV-APNC.

Concurrent validity based on head-to-head comparisons across indices was evaluated for the APNCUI [10, 12, 22–25], Kessner Index [10, 12, 22, 24, 25], GINDEX [12, 25], PHS/EPPC [12, 25], IPR/Prenatal Index [24, 25], Carvalho & Novaes Index [25], Ciari Index [25], Coutinho Index [25], and the Cluster solution [23]. Good concurrent validity was supported by moderate evidence for the APNCUI, the Kessner Index, GINDEX, PHS/EPPC, and IPR/Prenatal Index. Limited evidence supported good concurrent validity for the Carvalho & Novaes Index, the Ciari Index, the Coutinho Index, and the Cluster solution.

## Discussion

This systematic review identified 13 studies that reported on the measurement properties of 12 indices of prenatal care utilization. The APNCUI and the Kessner Index were described the most while others were evaluated in only one or two articles per index, which weakens the level of evidence for the results. We used the COSMIN checklist to evaluate their methodological quality and the level of evidence informing their uptake. The scientific literature has not comprehensively reported the measurement properties of commonly used indices of prenatal care utilization, and there is insufficient research to inform the choice of the best index. Most of the indices of prenatal care utilization currently used in research and clinical practice have been evaluated for at least some form of reliability and/or validity. Evidence about the responsiveness to change of these indices is absent from these evaluations. The indices of prenatal care utilization supported by the strongest evidence regarding their measurement properties were the APNCUI and the Kessner Index followed by the PHS/EPPC and the GINDEX. Moderate evidence informs the reliability, predictive and concurrent validity properties of the APNCUI and the Kessner Index. Decisions about their use should be supported on recommendations promoted by local prenatal care clinical practice guidelines (CPG). Both APNCUI and the Kessner Index have similar criteria for optimal timing of initiation of prenatal care (APNCUI 1–4 months; Kessner Index1–3 months) and number of prenatal care visits during pregnancy (APNCUI 11; Kessner Index 9 visits) and seem to align with current CPG recommendations made by ACOG. However, they have different category responses of prenatal care adequacy, with the APNCUI having an extra category of “Intensive” care during pregnancy that the Kessner Index does not consider. The discrepancy within the literature prevents a consensus being formed about the strongest index to measure the adequacy of prenatal care.

The most important strength of this systematic review is the use of the COSMIN taxonomy to evaluate the measurement properties of the proposed indices of prenatal care utilization based on the methodological qualities of the individual studies and the strength of the body of evidence that informs the use of each index. The use of COSMIN by two independent reviewers provided a consistent approach to assess the measurement properties of all indices.

One limitation of this review is that we did not include indirect evidence from studies in which the indices were actually applied either to measure prenatal care utilization as a predictor of pregnancy or birth outcomes, or as an outcome of any other risk factor. One important use of the indices of prenatal care utilization has been to evaluate policy or public interventions seeking to improve the organization and evaluation of prenatal care services. In such situations, the indices can be used to evaluate the changes in levels of prenatal care utilization of such interventions. It is yet to be determined if the utilization of prenatal care services translates into improvements in birth outcomes for the mother and child however, a number of these indices may be useful in examining population utilization levels.

Despite lingering uncertainty of the effectiveness of prenatal care and what adequacy entails, prenatal care has been proposed as a vital strategy to reduce the risk of adverse outcomes at delivery/birth [28]. Several studies have showcased the association between inadequate prenatal care and adverse pregnancy outcomes such as preterm birth, low birth weight, and neonatal death [29–33]. Ultimately, the lack of agreement on the best way to measure adequacy of prenatal care has important implications for tracking health care utilization during pregnancy and when formulating recommendations and policies about best practice.

Additionally, the indices are typically based on visit recommendations for average or low risk pregnancies and do not establish a recommended visit pattern for high risk women or for women with specific medical conditions. This may result in underestimating the prenatal care needs of high risk women and overestimating adequate utilization of prenatal care in the total population [12]. Broader exploration into other components of prenatal care in future research should serve to illuminate the underlining benefits from the care associated with adequate prenatal care.

Prenatal care utilization indices included in this review focus on quantifying the timing and amount of care used and therefore, they do not assess the quality or content of the prenatal services delivered [34]. Because these quantitative indices use data that are routinely collected in administrative health datasets or electronic medical records, they offer a viable alternative to develop audit indicators for quality improvement purposes, and to explore associations between adequacy of prenatal care and pregnancy and birth outcomes at a population level via observational research designs. Other questionnaires have been developed and validated to evaluate the content and quality of prenatal care based on women’s satisfaction and values [35]. There is a need to develop theoretically-grounded measures that are able to capture both quantitative and qualitative dimensions of prenatal care utilization and quality. Additional studies evaluating the validity, reliability and responsiveness of measures of prenatal care utilization are needed. A clear reporting of the procedures to measure measurement properties of the indices may facilitate their selection and use for clinical, surveillance, and research purposes.

Differences remain in the scientific literature and in CPG (e.g., ACOG, Society of Obstetricians and Gynaecologists of Canada) regarding recommendations about the timing and the frequency of prenatal care [1, 2]. However, what is common in these and other CPG is the notion that the frequency of prenatal care visits progressively increases with advancing gestation. Novel developments in screening tests during the first trimester of pregnancy and the notion that important complications that occur later in pregnancy can be predicted in the first trimester have recently reinforced the idea of having more visits at the beginning of pregnancy [36]. Finally, preconception care should be considered part of the spectrum of prenatal care, given that it is likely that many underlying comorbidities are identified prior to pregnancy. This has important implications for stratification of women into high and low risk groupings, which ultimately dictates an adequate frequency of prenatal care for the remainder of their pregnancy [37].

## Conclusion

Most commonly used indices of prenatal care utilization have moderate to limited evidence informing their validity and reliability. Current choices of a preferred index to measure prenatal care utilization can differ depending on the measurement properties that have priority to the users of the index. Important measurement properties such as criterion and predictive validity and responsiveness to change should be further evaluated for all the indices using sound research methodology.

## Supplementary information

**Additional file 1.** MEDLINE Search Strategy.

## Data Availability

Data sharing is not applicable to this article as no datasets were generated or analysed during the current study.

## Abbreviations

ACOG: American College of Obstetrics and Gynecology
APNCUI: Adequacy Perinatal Care Utilization Index
COSMIN: COnsensus-based Standards for the selection of health Measurement Instruments
CPG: Clinical Practice Guidelines
GINDEX: Graduated Prenatal Care Utilization Index
IQR: interquartile range
LV-APNC: Last Visit Adequacy of Prenatal Care Index
PHS/EPPC: US Public Health Service Expert Panel on Prenatal Care
PRISMA: Preferred Reporting Items for Systematic Reviews and Meta-Analyses
R-GINDEX: Revised-Graduated Prenatal Care Utilization Index
SOGC: Society of Obstetricians and Gynaecologists of Canada
