# Supplementary material for: Systematic review of the measurement properties of indices of prenatal care utilization
Source: BMC Pregnancy Childbirth. 2020 Mar 18;20:171. doi: 10.1186/s12884-020-2822-5 (PMC7079477; doi:10.1186/s12884-020-2822-5)
Supplement: Supplementary file 1 — Additional file 1. MEDLINE Search Strategy. [file 12884_2020_2822_MOESM1_ESM.docx]

**MEDLINE Search Strategy**

1. Prenatal Care/ or perinatal care/

2. ((pregnan* or prenatal or pre-natal or obstetric* or maternal or perinatal or peri natal) adj care).mp.

3. 1 or 2

4. (tool* or test* or inventor* or battery or profile or index or indices or scale* or instrument* or questionnaire*).mp.

5. measur*.ti.

6. measur*.ab. /freq=2

7. exp Questionnaires/

8. or/4-7

9. 3 and 8

10. Psychometrics/

11. "Reproducibility of Results"/

12. (reproducibil* or reliable or reliability or valid* or psychometr*).tw.

13. or/10-12

14. 9 and 13

15. (adequa* or utilzation or utilisation or supply or sufficient or access* or satisfaction or inadequa* or disparit* or quantity or quality or deficien* or complian*).mp.

16. 14 and 15

17. (Kessner or Adequacy of Prenatal Care Utilization or APNCU or APNCUI or Graduated Prenatal Care Utilization or GINDEX or Revised- Graduated Prenatal Care Utilization or R-GINDEX).mp.

18. 16 or 17
